# Dairy Products, Dietary Calcium and Bone Health: Possibility of Prevention of Osteoporosis in Women: The Polish Experience

**DOI:** 10.3390/nu5072684

**Published:** 2013-07-16

**Authors:** Lidia Wadolowska, Kamila Sobas, Justyna W. Szczepanska, Malgorzata A. Slowinska, Magdalena Czlapka-Matyasik, Ewa Niedzwiedzka

**Affiliations:** 1 Department of Human Nutrition, University of Warmia and Mazury, ul. Słoneczna 44a, Olsztyn 10-718, Poland; E-Mails: lidia.wadolowska@uwm.edu.pl (L.W.); justyna.szczepanska@uwm.edu.pl (J.W.S.); snake@moskit.uwm.edu.pl (M.A.S.); ewa.niedzwiedzka@wsfiz.edu.pl (E.N.); 2 Department of Hygiene of Human Nutrition, University of Life Sciences in Poznań, ul. Wojska Polskiego 31, Poznań 60-624, Poland; E-Mail: magdam@poczta.up.poznan.pl

**Keywords:** BMD, dairy products, dietary calcium, osteoporosis, women

## Abstract

The objective of the study was to analyze the consumption of dairy products and dietary calcium by women in the context of bone mineral density and to assess opportunities to prevent osteoporosis in a dietary manner. The study was carried out with 712 Polish women. In 170 women aged 32 to 59 bone mineral density (BMD) was measured. The data on the consumption of dairy products and dietary calcium and some other osteoporosis risk factors was collected from 712 women. The average calcium intake from a diet was 507 mg/day. Only 2% of the women met Polish calcium intake recommendations. During adulthood, dairy product consumption or dietary calcium intake did not differ significantly between women with low BMD (below −1 SD) and women with regular BMD (≥−1 SD) (47.4 *vs.* 44.3 servings/week and 459 *vs.* 510 mg/day, respectively, *p* > 0.05). The odds ratios adjusted for age, menstruation and BMI in women with upper BMD tercile in comparison to the reference group (bottom tercile) was 2.73 (95% CI: 1.14, 6.55; *p* < 0.05) for the daily consumption of dairy products during the pre-school period and 2.40 (95% CI: 1.01, 5.70; *p* < 0.05) for the daily consumption of dairy products during the school period. Two clusters of women were established. In the S1 cluster, low BMD (below −1 SD) was associated with older age (≥50 years), lack of menstrual cycle. In the S2 cluster, regular BMD (≥−1 SD) was related to younger aged women (<50 years), presence of menstrual cycle, consumption of higher level of dairy products (≥28 servings/week) during adulthood and daily intake of dairy products during childhood and adolescence. The results indicate that good bone health to the large extent depended upon the combined impact of dietary factors and some non-modifiable risk factors of osteoporosis such as age and the presence of menstruation. Consumption of dairy products in childhood and adolescence may improve bone mineral density and reduce the risk of osteoporosis in adult women.

## 1. Introduction

Osteoporosis presents a growing health problem in many countries around the world [1]. It is one of the most common complaints in people aged 50 and over and affects 30% of women and 8% of men in this age group [2]. In Europe, USA and Japan, osteoporosis has been diagnosed in 75 million individuals [1,2]. In Europe, the annual costs of treatment of osteoporotic fractures amount to 25 billion Euros [1]. Due to the aging of the society the incidence of osteoporosis will increase and thus it will generate a still further increase in treatment costs.

Women over the world are more exposed to osteoporosis and osteoporotic fractures then men [1,2,3,4]. In Europe and USA as well as Poland approximately 30% of postmenopausal women have osteoporosis of which 40% will sustain one or more fragility fracture in their remaining lifetime [3,4]. In Poland, osteoporosis affects 7% of women aged 45–64, 25% of women aged 65–74 and over 50% of women older than 75 years [2]. In 2010, the number of cases of osteoporotic fractures in people aged 50 and over was 2.7 million, including 2.2 million females and 0.5 million males [3]. It is predicted that four out of ten Polish women will be affected with at least one vertebral fracture before turning 80 years of age [3]. Post-fracture complications result in the annual mortality of 20% of women and 30% of men with femoral neck fractures and a further 30% require constant medical care. It is thus justifiable for health science professionals to have an interest in the early diagnosis of osteoporosis, by understanding the risk factors and taking preventive measures.

Osteoporosis is a systemic metabolic bone disease which presents reduction in bone density together with abnormal structure [5,6]. Asymptomatic and pathological fractures are frequently the first clinical symptoms. Reduction in bone mineral density (BMD) with age is generally unavoidable. In the age range from 20 to 79, the proportion of individuals with regular bone mass and BMD values decreases in a mode near to linear, while the incidence of osteoporosis increases linearly [7]. Until recently, diagnosis and treatment of osteoporosis was based on bone densitometry. Currently, both the diagnosis and therapy of osteoporosis focus on determining the individual risk for bone fracture [3,8]. However, this approach requires collecting information on the occurrence of dietary and non-dietary risk factors for bone fractures. Non-dietary factors increasing bone fracture risk include female sex, white race, advanced age, menstruation disorders, early menopause, previous fractures, hip fractures in parents, low BMI, low bone mass, administration of corticosteroids, rheumatoid arthritis, limited physical activity, poor self-evaluated health, smoking and alcohol abuse [3,5,6]. Since some of these elements are non-modifiable risk factors (e.g., age, sex, inheritance), then more attention should be paid to the modifiable risk factors for bone fractures.

Dietary risk factors for bone fractures play a special role in the prevention of osteoporosis. Numerous studies indicate that adequate calcium intake improves bone mineral density [9,10,11]. However, the role of dietary calcium and dairy products in preventing osteoporosis has not yet been fully explained. It is commonly thought that adequate intake of calcium during childhood and adolescence is most important [12,13]. It leads to high peak bone mass which serves as a sort of “deposit” for subsequent years of life [14,15]. The impact of calcium intake and consumption of dairy products on bone density in later stages of life is less understood [10,16]. It is thought that in adulthood, the role of dietary factors may be less and may mainly influence the rate of bone loss. It has been estimated that in post-menopausal women the involution rate of bone loss may change over a wide range of values from 1% to 5% or more per year [7].

The predicted increase in the incidence of osteoporosis in women justifies a search for opportunities to prevent bone fractures. Undoubtedly, attention should be focused on exploring modifiable dietary risk factors. An in-depth understanding of these elements will provide opportunities for construction and implementation of prevention programs and improvement of public health. The objective of the studies was to analyze the consumption of dairy products and dietary calcium by women in the context of bone mineral density and to assess opportunities to prevent osteoporosis. We aimed to ascertain if there is a possibility to provide good bone status in the dietary manner, without nutritional intervention, but with the usual intake of dairy products and dietary calcium, typical of a Polish diet.

## 2. Methods

### 2.1. Sample Selection

The studies were carried out within the scientific project “Analysis of mother-daughter dairy products food patterns in relation to bone mineral status and calcium deficiency and osteoporosis risk among women. MODAF Study” financed by The Polish Ministry of Science and Higher Education. Mother-daughter pairs were recruited within the framework of these studies. This publication presents the results that relate to these women. Permission for the research was obtained from the Bioethics Committee of the Faculty of Medical Sciences, University of Warmia and Mazury in Olsztyn on 17 June 2010, Resolution No. 20/2010.

The sample was collected with snowball sampling by means of personal contacts and announcements. The recruitment was carried out in the towns and villages in central and north-eastern Poland. During the recruitment process, women and girls were informed about the subject and the purpose of the studies and, thus, a self-selection of respondents occurred. Women/girls were excluded if they consumed a non-dairy diet because of allergies or lactose intolerance. The participation in the study required consent given by both mother and daughter. The inclusion criteria for mother and daughter were: lack of nutritional disorders, lack of professional sporting activity on an advanced level and lack of diseases, surgical procedures or administration of drugs (for longer than a year) that might disrupt the hormonal balance, metabolism or bone metabolism and additionally for the mother’s age under 60 years [7].

The study included 712 women aged 29 to 59 (on average 43.8 years of age) and a subsample of 170 women (24% of total sample) aged 32 to 59 (on average 45.5 years; Table 1). In the subsample bone mineral density (BMD) was measured and the data on the consumption of dairy products and dietary calcium and some other osteoporosis risk factors was collected. For organizational reasons for the subsample all mothers and daughters living in one typical area of Poland were chosen. This was located in the central part of the country (Swietokrzyski region). The data were collected in the homes of the respondents, and limiting the area gave a lower index of participants refusing and made densitometer transportation easier. The women from the total sample and in the subsample (with bone densitometry testing) did not differ in social and demographic parameters (Table 1).

**Table 1:** Description of the total sample and sub-sample of women tested with bone densitometry.

Parameters	Sample percentage (%)	
	Total sample	Sub-sample
Sample size	712	170
Age# (years)	43.8 ± 5.8 (29–59)	45.5 ± 5.8 (32–59)
BMI# (kg/m^2^)	25.6 ± 4.2 (16–44)	26.2 ± 4.7 (18–44)
Education		
elementary	5	1
secondary	67	71
higher	28	28
Place of living		
village	48	63
town < 50,000 residents	16	6
town 50,000–100,000 residents	14	11
city > 100,000 residents	22	20
Self-declared economic situation		
bad	1	1
satisfactory	23	29
good	66	63
very good	10	7
Description of household		
We do not have enough resources even for the cheapest food and clothing	0	1
We do not have enough resources for housing fees	1	2
We have enough resources only for food and clothing	7	8
We live very thriftily	14	13
We live relatively thriftily	54	51
We can afford everything without limitations	23	25

# mean ± standard deviation; () in the brackets indicated the minimum-maximum range.

### 2.2. General Information

All information was collected by means of “face-to-face” contact. The investigations and measurements were conducted by well-trained researchers.

During the direct surveys, information on risk factors influencing bone mass and presence of osteoporosis was collected. Based on the data available in the literature, a comprehensive list of dietary and non-dietary risk factors was prepared. In total, information regarding 22 risk factors was collected [3,8,16]. This paper presents the selected risk factors associated with BMD and interest related to the paper objective. Finally our paper included six dietary risk factors (dairy products intake, calcium intake, consuming calcium-enriched food, taking calcium supplements, consuming daily dairy products during the pre-school period, consuming daily dairy products during school period) and three non-dietary risk factors well known as confounders, influencing bone mineral density (age, if menstruating, body mass index) [3,8,16].

To characterize the respondents information regarding their socio-economic situation was collected (Table 1). The women were asked about their date of birth (to calculate their age) and education level (they could choose “elementary”, “secondary” or higher” answers). For a better description of a woman’s economic situation two closed-questions were used:

– self-declared economic situation with four answers: “bad”, “satisfactory”, “good” and “very good”,
– self-declared situation of household with six answers: “We do not have enough resources even for the cheapest food and clothing”, “We do not have enough resources for housing fees”, “We have enough resources only for food and clothing”, “We live very thriftily”, “We live relatively thriftily” and “We can afford everything without limitations”.

Most women declared a good economic situation (63%), 1% of them had a bad economic situation and 7% of them had very good economic situation (Table 1).

The respondents were asked about taking calcium supplements, daily consumption of dairy products during the pre-school period and school period and the consumption of calcium-enriched food. The other questions asked separately concerned consumption of calcium-enriched juices and calcium-enriched ready-to-eat cereals. Consumption of calcium-enriched juices or calcium-enriched ready-to-eat cereals was classified as “consumption of calcium-enriched food”. For all the questions mentioned above, the respondents chose between “yes”, “no” and “don’t know” answers.

Furthermore, the women were asked about their menstrual cycle (yes, regularly; yes, irregularly; the menstrual cycle has not yet started, the menstrual cycle has ceased).

### 2.3. Assessment of Consumption of Dairy Products and Dietary Calcium

The consumption of calcium in a daily diet (DD) was determined with a semi-quantitative method which measured food consumption frequency based on the intake of calcium in dairy products. A validated ADOS-Ca questionnaire was used to evaluate the consumption of dairy products [17]. The data was collected on the frequency of consumption and portion size usually consumed (during the last six months) of 11 groups of dairy products: milk (in beverages and soups), rennet cheese, cottage cheese, processed cheese, natural and fruit yoghurt, buttermilk/fermented milk, sour cream/cream, ice cream during and after the season, cream cheese, and fromage-type cheese. Respondents were questioned about typically eaten servings. Eight categories of consumption frequency were calculated into the average consumption frequency (times/day) with the use of consumption frequency indices determined during the validation of the questionnaire (indices are given in brackets) [17]:

– never (0),
– less seldom than once a week (1/30),
– once-twice a week (1/7),
– 3–4 times a week (3/7),
– 5–6 times a week (5/7),
– once daily (1),
– twice daily (2),
– 3 times a day (3).

The average consumption of dairy products (g/day), average consumption of calcium in dairy products (mg/day) and average consumption of calcium in DD (mg/day) for each person was calculated with tables of food nutritional values [18] and a regression equation [17]. Calcium intake from each product was calculated on the basis of formula (1), e.g., calcium intake from milk:

Ca_milk_ = *a*_milk_ × (*b*_milk_ × *c*_milk_/100) (1)

where:

Ca_milk_—calcium intake from milk (mg/person/day),
*a*_milk_—product intake frequency index, e.g., milk,
*b*_milk_—the amount of product in single consumption, e.g., milk (g),
*c*_milk_—calcium content in 100 g of product, e.g., milk (mg/100 g).

Total calcium intake from dairy products was calculated according to formula (2), adding the calcium intake from all 11 groups of dairy products:

Ca_dairy products_ = Ca_milk in beverages_ + Ca_milk soup_ + Ca_fruit yoghurt_ +…+ Ca_cream_ (2)

Finally, the calcium intake from the daily diet (Ca_DD_) was calculated on the basis of calcium intake from dairy products (Ca_dairy products_) according to formula (3) [17].

Ca_DD_ = 338.21 + 0.61 Ca_dairy products_ (3)

The number of women (in %) who met the Polish calcium intake recommendations and did not meet calcium intake recommendations was calculated [19] using the probability method. The established cut-off points (*i.e*., *z*-values of individual calcium intake <−1 SD or >1 SD) produced conclusions with a probability of 0.85 [19]. Between <−1 SD and >1 SD there is an area of calcium intake interpreted as “neither meets nor does not meet the calcium intake recommendations” [19]. Finally, we assessed the intake of calcium in 13% of women which produced difficulties in statistical analysis.

In order to better assess and interpret the results, the division into >400 mg/day and ≤400 mg/day for calcium consumption in the daily diet and into ≥28 servings/week and <28 servings/week for dairy product consumption was implemented. The cut-off value for calcium consumption was assumed based on literature reviews [16,20] and Polish calcium intake recommendations [21]. The cut-off for calcium intake (400 mg/day) is equal to half the amount of calcium per day (EAR) recommended for Polish women aged 19–49 years [21]. Another reason to choose this cut-off was low calcium intake in women (mean value about 500 mg/person/day). The cut-off value for dairy products was established on the basis of distribution analysis of dairy products consumption. Many experts recommend consumption of 2–3 servings of dairy products a day for adults (14–21 servings/week) [22]. In our sample and sub-sample we stated relatively high dairy product consumption in women (mean value above 44 servings/week). Because of this we decided to choose as a cut-off for dairy products 28 servings/week (equivalent to 4 servings/day). One serving of dairy products was an equivalent of about 300 mg calcium, e.g., 250 g of milk, 200 g of natural or fruit yoghurt, 300 g of fermented milk, 300 g (1 cup) of fresh cheese, 40 g (2 slices) of rennet cheese, 210 g (3 spoons) of ice cream, 300 g of homogenized cheese, 80 g of processed cheese (1/2 cup) [22,23].

For better results the presentation of 11 groups of dairy products were aggregated into five groups:

– milk (in beverages and soups),
– cheese: rennet cheese, fromage-type cheese, processed cheese,
– yoghurt: natural yoghurt, fruit yoghurt, buttermilk/fermented milk,
– fresh cheese: cottage cheese, cream cheese,
– cream: sour cream/cream, ice cream.

There were no differences (*p* < 0.05) between the women from the total sample (*N* = 712) and the women from the sub-sample (*N* = 170) in dairy product consumption in total (44.3 *vs.* 44.5 servings/week) as well as each item of dairy product, in taking calcium supplements (16% *vs.* 22%, respectively), daily consumption of dairy products during pre-school period (63% *vs.* 64%, respectively) and daily consumption of dairy products during school period (57% *vs.* 61%, respectively; Appendix A). More women from the sub-sample than women from the total sample consumed calcium-enriched food (89% *vs.* 78%, respectively; *p* < 0.05).

### 2.4. Assessment of Bone Mineral Density and Body Mass

Bone mineral density (BMD) was measured with a pDEXA densitometer with a dual-energy X-ray absorptiometry (DXA) and expressed as a *T*-score index, *i.e*., number of standard deviations related to peak bone mass in young adults [6]. The measurement was taken in the distal part of the radius and the ulna. The women were divided into groups with different BMD:

- BMD < −1 SD—interpreted as low bone mineral density (osteopenia or osteoporosis),
- BMD ≥ −1 SD—interpreted as regular bone mineral density.

Only a few women had low bone mineral density. Because of this we established three other groups of women according to their BMD. Based on the analysis of the distribution of bone mineral density, the women with low, medium and high mineral bone density were classified. Three subgroups were created in accordance with the BMD tercile ranges (Table 2):

- T1BMD: <352.2 mg/cm^2^—bottom BMD tercile,
- T2BMD: 352.2–401.1 mg/cm^2^—middle BMD tercile,
- T3BMD: >401.1 mg/cm^2^—upper BMD tercile.

The T1BMD women (47.1 years of age) were older than the T2BMD women (44.3 years of age). The age of the T3BMD women (45.3 years of age) did not differ statistically from the age of the T1BMD and T2BMD women (Table 2).

**Table 2:** Description of the sample depending on bone mineral density (BMD).

Parameters	Total	Bone mineral density		Bone mineral density		
		BMD < −1 SD	BMD ≥ −1 SD	T1BMD	T2BMD	T3BMD
Sample size	170	12	158	57	57	56
Sample percentage (%)	100	7	93	33	33	33
Age# (years)	45.5 ± 5.8	50.9 ± 4.5 ^a^	45.1 ± 5.7 ^a^	47.1 ± 6.8 ^b^	44.3 ± 5.0 ^b^	45.3 ± 5.3
BMD# (mg/cm^3^)	379.2 ± 59.1 (218–615)	275.1 ± 22.3 (218–299)	387.1 ± 53.2 (302–615)	321.6 ± 29.1 (218–351)	373.8 ± 15.2 (352–401)	443.4 ± 44.5 (402–615)

# mean ± standard deviation; T1BMD, T2BMD, T3BMD—terciles of bone mineral density; () in the brackets indicated the minimum-maximum range; ^a,b^ significance of differences at *p* < 0.05.

Body weight and height of the respondents were measured and body mass index was calculated (BMI, kg/m^2^). For BMI, the WHO criteria were applied and the percentage of overweight (BMI ≥ 25 kg/m^2^) and non-overweight women (BMI < 25 kg/m^2^) was assessed [24]. The average BMI was 26.2 kg/m^2^ (Table 1).

### 2.5. Statistical Analysis

The average intake of calcium and consumption of dairy products, age of women, BMI, and BMD were expressed with the mean (x) and the standard deviation (SD). For the selected parameters, the minimum-maximum range or 95% confidence interval (95% CI) was given. The average consumption of calcium and dairy products by women with different bone mineral density was compared with the Kruskal-Wallis test [25]. The distributions of parameters were compared with Pearson’s chi-square test. The test for structure indices was used to compare the percentage of individuals in pairs.

The impact of individual risk factors on bone mineral density was evaluated with logistic regression [26,27]. All the used paper risk factors of osteoporosis were included in the logistic regression (six dietary risk factors and three non-dietary confounders). For the purpose of this analysis, the division into two categories was implemented for each osteoporosis risk factor. The participants were classified as:

– aged: <50 years, ≥50 years,
– menstruation: yes, no,
– with BMI: <25 kg/m^2^, ≥25 kg/m^2^,
– consuming dietary calcium at: >400 mg/day, ≤400 mg/day,
– consuming dairy products: ≥28 servings/week, <28 servings/week,
– consuming calcium-enriched food: yes, no,
– taking calcium supplements: yes, no,
– every day consumption of dairy products during pre-school period: yes, no,
– every day consumption of dairy products during school period: yes, no.

The reference group (OR = 1.00) included the individuals with low BMD, *i.e.*, BMD < −1 SD or T1BMD (bottom BMD tercile) in two separately made analyses. Wald's chi-square statistics was used to determine the significance of the impact of risk factors on the presence of low bone mineral density. For the six dietary risk factors the ORs adjusted for age, menstruation and BMI (separately and together) were calculated [26,27].

The relationship between BMD and osteoporosis risk factors in the complex system was evaluated with a correspondence analysis. We used Burt’s panels [28]. The correspondence analysis is a qualitative multifactorial analysis. It provides information about the structure of the connections between columns and rows of multiple divided tables [28]. It makes possible the clearly graphic presentation of the relation between categories of qualitative variables. It finely completes and explains relationships which are revealed in multiple divided tables. Typically the results of the correspondence analysis are presented in a figure in the system of two coordinates—dimension 1 and dimension 2 (similar to *X* and *Y* axis) because of better presentation on the plane. On the figure are placed points corresponding to each feature category. The correspondence analysis takes into consideration distance between points and their position in multi-dimensional space, although the graphic picture is presented on the plane for simplicity. The mutual relation between category variables is bigger the lower the distance between points and the bigger the explained level of inertia. The inertia of the system can be interpreted similarly to variance–the bigger the inertia the better is the described relation between variables and their categories.

At first into the correspondence analysis BMD (<−1 SD and ≥−1 SD), six dietary and three non-dietary risk factors (all used in the paper) were included. Next we excluded risk factors from the correspondence analysis one by one. We assessed the explained level of inertia and chose the best model with the higher explained inertia. Finally the correspondence analysis included BMD and five risk factors with 12 categories in total, after excluding “calcium intake”, “consuming calcium-enriched food”, “taking calcium supplements” and “BMI” because they lowered the explained level of inertia:

– age: <50 years, ≥50 years,
– menstruation: yes, no,
– consumption of dairy products: ≥28 servings/week, <28 servings/week,
– every day consumption of dairy products during pre-school period: yes, no,
– every day consumption of dairy products during school period: yes, no,
– bone mineral density: low BMD (<−1 SD), regular BMD (≥−1 SD).

We obtained a high level of explained inertia in this model. In the same way we analyzed the relation between BMD divided into terciles (T1BMD, T2BMD, T3BMD) and six dietary and three non-dietary risk factors (all used in the paper), but all the next models were worse than the model described above.

After dividing the sample for menstruation and no menstruation in women or regarding women’s age groups (29–39, 40–49 and 50–59 years), no differences in BMD or *T*-sore BMD were found (Appendix B). Because of this we decided not to prepare the analyses separately for menstruation and no menstruation in women. The statistical analysis was performed with Statistica 10.0 PL software (StatSoft, Kraków, Poland).

## 3. Results

### 3.1. Consumption of Dairy Products and Dietary Calcium Intake in Relation to BMD

The average consumption of dairy products by women was 44.5 servings/week (Table 3). The most commonly-consumed dairy products included milk, cheese and yoghurt. The study did not find any difference in the consumption of dairy products in women with different BMD. Dairy product consumption by women with low BMD (below −1 SD) was 47.4 servings/week and women with regular BMD was 44.3 servings/week (Table 3).

The average intake of dietary calcium by women was 507 mg/day and did not differ significantly between the groups with different BMD (Table 3). Dietary calcium intake by women with low BMD (below −1 SD) was 459 mg/day and women with regular BMD was 510 g/day. The main sources of calcium in the diet included milk, cheese and yoghurt, which provided 67% of the total amount of calcium. Among women with different BMD there were minor differences in calcium intake from dairy products. The T3BMD women consumed less calcium from cheese in comparison with the T1BMD women (81 *vs.* 120 mg/day, respectively).

The women who met Polish calcium intake recommendations was 2% in total (Table 3). More women with regular BMD (≥−1 SD) met calcium intake recommendations than women with low BMD (<−1 SD), 2% *vs.* 0%, respectively, but differences were not statistically significant (*p* >0.05). More women T3BMD met calcium intake recommendations than women T2BMD, 5% *vs.* 0%, respectively (*p* < 0.05).

### 3.2. Presence of Risk Factors for Osteoporosis in Relation to BMD

The factors that had a positive impact on bone mineral density with the highest presence included: consumption of dairy products ≥28 servings/week (88% of the sample in total), consumption of calcium-enriched food (89%), regular menstrual cycles (82%), age < 50 years (78%) and daily consumption of dairy products during pre-school and school periods (64% and 61% respectively; Table 4). The beneficial behavior that reduced the risk of osteoporosis in a higher number of women with BMD ≥ −1 SD than in women with BMD < −1 SD were reported for two non-modifiable risk factors. More women with BMD ≥−1 SD in comparison with women with BMD < −1 SD were aged < 50 years (80% *vs.* 50%; *p* < 0.05) and regular menstruation (85% *vs.* 42%; *p* < 0.05).

**Table 3:** Consumption of dairy products and calcium from dairy products and daily diet (DD) in relation to bone mineral density (BMD) in women (mean ± standard deviation).

Dairy products	Total	Bone mineral density		*p*-value for Kruskal-Wallis test	Bone mineral density			*p*-value for Kruskal-Wallis test
		BMD < −1 SD	BMD ≥ −1 SD		T1BMD	T2BMD	T3BMD	
Sample size	170	12	158		57	57	56	
**Dairy products (servings/week) §**								
Milk	12.4 ± 6.3	12.8 ± 8.0	12.4 ± 6.1	ns	12.8 ± 7.0	11.9 ± 5.9	12.5 ± 5.9	ns
Cheese	11.2 ± 5.6	13.7 ± 6.2	11.1 ± 5.5	ns	12.1 ± 5.8	10.9 ± 5.4	10.7 ± 5.7	ns
Yoghurt	13.4 ± 7.0	12.9 ± 8.5	13.4 ± 6.9	ns	13.9 ± 7.1	12.6 ± 6.8	13.5 ± 7.3	ns
Fresh cheese	5.4 ± 3.0	5.8 ± 2.3	5.3 ± 3.0	ns	5.4 ± 3.2	5.7 ± 3.0	5.0 ± 2.7	ns
Cream	2.2 ± 1.2	2.3 ± 1.8	2.2 ± 1.2	ns	2.2 ± 1.2	2.1 ± 1.4	2.2 ± 1.1	ns
Dairy products in total	44.5 ± 14.0	47.4 ± 17.0	44.3 ± 13.8	ns	46.4 ± 15.5	43.1 ± 12.0	44.0 ± 14.3	ns
**Calcium from dairy products (mg/day)**								
Milk	124 ± 174	78 ± 84	127 ± 179	ns	111 ± 135	108 ± 137	153 ± 233	ns
Cheese	93 ± 129	106 ± 93	92 ± 131	ns	120 ± 158 ^a^	79 ± 105	81 ± 114 ^a^	ns
Yoghurt	122 ± 159	120 ± 139	122 ± 161	ns	132 ± 145	117 ± 127	118 ± 199	ns
Fresh cheese	24 ± 25	28 ± 37	23 ± 24	ns	24 ± 27	25 ± 21	22 ± 27	ns
Cream	12 ± 19	9 ± 13	12 ± 19	ns	10 ± 12	13 ± 24	13 ± 19	ns
Calcium from dairy products in total	375 ± 269	340 ± 252	378 ± 270	ns	396 ± 269	341 ± 206	386 ± 321	ns
Calcium from DD	507 ± 363	459 ± 340	510 ± 365	ns	536 ± 363	461 ± 279	522 ± 433	ns
Women who met # calcium intake recommendations (%)	2	0	2	ns *	0	0 ^b^	5 ^b^	ns *
Women who did not meet # calcium intake recommendations (%)	11	17	10	ns *	12	9	11	ns *

§ One serving of dairy products was an equivalent of about 300 mg calcium; # used cut-off points produced conclusions with a probability of 0.85 and because of this the number of women who met and did not meet calcium intake recommendations did not sum up to 100%; T1BMD, T2BMD, T3BMD—terciles of bone mineral density; ^a,b^ significance of differences at *p* < 0.05; ns—non significant differences at *p* < 0.05; * *p* for chi^2^ test.

**Table 4:** Distribution of the sample and the odds ratio (OR) for risk factors for osteoporosis in bone mineral density (BMD) women groups.

Parameters	Total	Bone mineral density				
		BMD < −1 SD	BMD ≥ −1 SD	T1BMD	T2BMD	T3BMD
Sample size	170	12	158	57	57	56
**Age <** **50 years**						
Number of cases	132	6	126	39	48	45
Percentage of cases (%)	78	50 ^a^	80 ^a^	68 ^b^	84 ^b^	80
OR (95% CI)		1.00	3.94 * (1.17, 13.14)	1.00	2.46 (0.97, 6.14)	1.89 (0.79–4.52)
**Menstrual cycle**						
Number of cases	139	5	134	41	53	45
Percentage of cases (%)	82	42 ^c^	85 ^c^	72 ^d^	93 ^d,e^	80 ^e^
OR (95% CI)		1.00	7.82 ** (2.27, 26.90)	1.00	5.17 * (1.59, 16.90)	1.60 (0.66, 3.90)
**BMI <** **25** **kg/m^2^**						
Number of cases	67	6	61	22	26	19
Percentage of cases (%)	39	50	39	39	46	34
OR (95% CI)		1.00	0.63 (0.19, 2.06)	1.00	1.33 (0.63, 2.84)	0.82 (0.38, 17.80)
**Consumption of dietary calcium >** **400** **mg/day**						
Number of cases	90	8	82	37	27	26
Percentage of cases (%)	53	67	52	65 ^f,g^	47 ^f^	46 ^g^
OR (95% CI)		1.00	0.54 (0.15, 1.88)	1.00	0.49 (0.23, 1.04)	0.47 (0.20, 1.01)
Age-adjusted OR (95% CI)		1.00	0.61 (0.17, 2.23)	1.00	0.50 (0.23, 1.09)	0.45 * (0.21, 0.97)
OR (95% CI) †		1.00	0.54 (0.15, 1.88)	1.00	0.51 (0.23, 1.12)	0.47 (0.22, 1.03)
OR (95% CI) #		1.00	0.60 (0.10, 3.44)	1.00	0.48 (0.23, 1.04)	0.48 (0.22, 1.05)
OR (95% CI) §		1.00	0.62 (0.16, 2.36)	1.00	0.50 (0.23, 1.11)	0.47 (0.21, 1.05)
**Consumption of dairy products ≥28 servings/week**						
Number of cases	150	10	140	51	49	50
Percentage of cases (%)	88	83	89	89	86	89
OR (95% CI)		1.00	1.56 (0.31, 7.76)	1.00	1.39 (0.44, 4.34)	1.02 (0.29, 3.54)
Age-adjusted OR (95% CI)		1.00	1.25 (0.24, 6.54)	1.00	1.61 (0.49, 5.25)	1.23 (0.35, 4.29)
OR (95% CI) †		1.00	1.53 (0.28, 8.33)	1.00	1.54 (0.46, 5.17)	1.07 (0.32, 3.65)
OR (95% CI) #		1.00	1.55 (0.31, 7.72)	1.00	1.36 (0.43, 4.27)	1.04 (0.3, 3.52)
OR (95% CI) §		1.00	1.36 (0.23, 7.88)	1.00	1.52 (0.45, 5.12)	1.26 (0.36, 4.44)
**Consumption of calcium-enriched food**						
Number of cases	151	11	140	53	50	48
Percentage of cases (%)	89	92	89	93	88	86
OR (95% CI)		1.00	0.71 (0.08, 5.90)	1.00	0.54 (0.14, 1.98)	0.45 (0.13, 1.62)
Age-adjusted OR (95% CI)		1.00	0.53 (0.06, 4.83)	1.00	0.45 (0.12, 1.75)	0.45 (0.12, 1.63)
OR (95% CI) †		1.00	0.62 (0.06, 6.34)	1.00	0.41 (0.10, 1.70)	0.45 (0.12, 1.61)
OR (95% CI) #		1.00	0.55 (0.06, 5.19)	1.00	0.53 (0.14, 1.95)	0.47 (0.13, 1.67)
OR (95% CI) §		1.00	0.64 (0.07, 5.87)	1.00	0.40 (0.10, 1.68)	0.46 (0.13, 1.70)
**Taking calcium supplement **						
Number of cases	38	3	35	14	12	12
Percentage of cases (%)	22	25	22	25	21	21
OR (95% CI)		1.00	0.85 (0.22, 3.36)	1.00	0.82 (0.34, 1.99)	0.83 (0.34, 2.04)
Age-adjusted OR (95% CI)		1.00	0.95 (0.23, 3.96)	1.00	0.84 (0.34, 2.08)	0.90 (0.36, 2.21)
OR (95% CI) †		1.00	0.94 (0.22, 3.95)	1.00	0.83 (0.33, 2.10)	0.85 (0.35, 2.08)
OR (95% CI) #		1.00	0.68 (0.07, 6.14)	1.00	0.79 (0.32, 1.94)	0.81 (0.33, 1.98)
OR (95% CI) §		1.00	0.75 (0.17, 3.28)	1.00	0.80 (0.31, 2.03)	0.86 (0.35, 2.14)
**Daily consumption of dairy products during pre-school period**						
Number of cases	109	7	102	34	33	32
Percentage of cases (%)	64	58	65	60 ^h^	58 ^i^	75 ^h,i^
OR (95% CI)		1.00	1.30 (0.39, 4.33)	1.00	0.93 (0.44, 1.98)	2.03 (0.90, 4.57)
Age-adjusted OR (95% CI)		1.00	1.50 (0.44, 5.04)	1.00	1.10 (0.50, 2.42)	2.51 * (1.07, 5.91)
OR (95% CI) †		1.00	2.19 (0.58, 8.32)	1.00	1.26 (0.56, 2.80)	2.34 ** (1.01, 5.44)
OR (95% CI) #		1.00	1.88 (0.36, 9.86)	1.00	0.93 (0.44, 1.98)	2.07 (0.91, 4.68)
OR (95% CI) §		1.00	4.01 (0.86, 18.63)	1.00	1.26 (0.56, 2.81)	2.73 * (1.14, 6.55)
**Daily consumption of dairy products during school period**						
Number of cases	104	8	96	35	27	42
Percentage of cases (%)	61	67	61	61 ^j,k^	47 ^j,l^	75 ^k,l^
OR (95% CI)		1.00	0.77 (0.22, 2.71)	1.00	0.57 (0.27, 1.20)	1.88 (0.83, 4.26)
Age-adjusted OR (95% CI)		1.00	1.05 (0.28, 3.92)	1.00	0.63 (0.29, 1.36)	2.11 (0.92, 4.89)
OR (95% CI) †		1.00	1.05 (0.28, 3.91)	1.00	0.71 (0.32, 1.57)	2.02 (0.88, 4.62)
OR (95% CI) #		1.00	1.64 (0.31, 8.60)	1.00	0.56 (0.27, 1.20)	2.01 (0.88, 4.62)
OR (95% CI) §		1.00	1.22 (0.31, 4.83)	1.00	0.71 (0.32, 1.56)	2.40 * (1.01, 5.70)

† adjusted for menstruation; # adjusted for BMI; § adjusted for age, BMI and menstruation; () in the brackets indicated 95% confidence interval; T1BMD, T2BMD, T3BMD—terciles of bone mineral density; ^a-a,…,l-l^ significance of differences at *p* < 0.05; significance of differences for OR at: * *p* < 0.05, ** *p* < 0.01.

The division of women into tercile groups according to their BMD significantly differentiated the distribution of six risk factors for osteoporosis (Table 4). The significance differences were found in women’s age, menstrual cycle, consumption of dietary calcium at >400 mg/day, consumption of dairy products at ≥28 servings/week and every day consumption of dairy products during pre-school and school periods. More T3BMD women than T2BMD women or T1BMD women consumed dairy products every day during the pre-school period (75% *vs.* 58% or 60%; *p* < 0.05) and consumed dairy products every day during the school period (75% *vs.* 47% or 61%; *p* < 0.05). More T2BMD women than T1BMD women were <50 years of age (84% *vs.* 68%; *p* < 0.05). More T2BMD women than T1BMD women or T3BMD women had regular menstrual cycles (93% *vs.* 72% or 80%; *p* < 0.05), whereas more T1BMD women than T2BMD women and T3BMD women consumed dietary calcium >400 mg/day (65% *vs.* 47% and 46%; *p* < 0.05).

The odds ratio for regular BMD (≥−1 SD) in comparison to low BMD (<−1 SD) was 3.94 (95% CI: 1.17, 13.14; *p* < 0.05) in women aged <50 years and 7.82 (95% CI: 2.27, 26.90; *p* < 0.01) for women with a menstrual cycle (Table 4). In comparison with the bottom tercile BMD (reference group), the odds ratio for T2BMD (middle tercile) women with a menstrual cycle was 5.17 (95% CI: 1.59, 16.90; *p* < 0.05).

The odds ratio adjusted for age, menstruation and BMI for regular BMD (≥−1 SD) in comparison to low BMD (<−1 SD) was 4.01 for the daily consumption of dairy products during pre-school period, 1.36 for consumption of dairy products ≥28 servings/week, 1.22 for the daily consumption of dairy products during the school period, 0.75 for taking a calcium supplement, 0.64 for consumption of calcium enriched food and 0.62 for consumption of dietary calcium at >400 mg/day (Table 4). All the above mentioned odds ratios were not statistically significant (*p* > 0.05).

Two odds ratios adjusted for age, menstruation and BMI in women with T3BMD (upper tercile) in comparison to the reference group (bottom BMD tercile) were statistically significant and amounted to 2.73 (95% CI: 1.14, 6.55; *p* < 0.05) for the daily consumption of dairy products during the pre-school period and 2.40 (95% CI: 1.01, 5.70; *p* < 0.05) for the daily consumption of dairy products during the school period (Table 4). In T3BMD women in comparison with T1BMD women the odds ratio adjusted for age, menstruation and BMI was 1.26 for consumption of dairy products ≥28 servings/week, 0.86 for taking calcium supplement, 0.47 for the consumption of dietary calcium at >400 mg/day and 0.46 for consumption of calcium enriched food—these ORs were outside the range of statistical significance.

**Figure 1:**
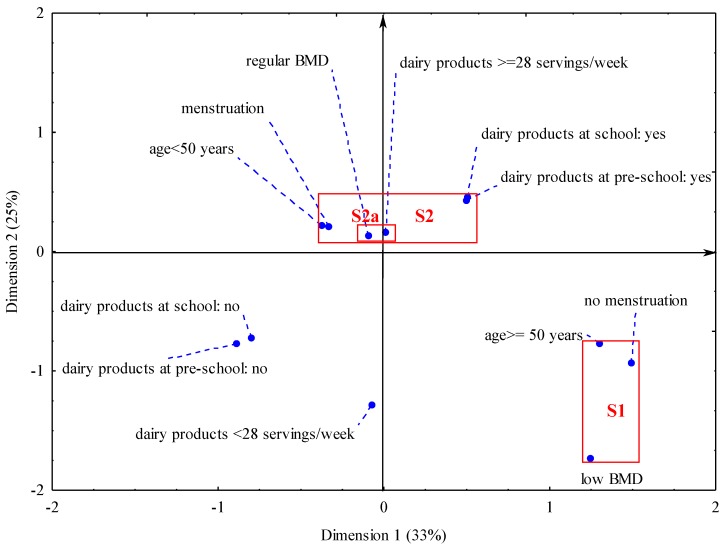
Graphical presentation of the relationship between risk factors for osteoporosis and bone mineral density in women. () in brackets are the given explained inertia in two dimensions; areas marked by rectangles and signed S1 and S2 point two clusters grouping features correlated with each other; Notes: “dairy products ≥ 28 servings/week”—consumption of dairy products ≥ 28 servings/week; “dairy products <28 servings/week” —consumption of dairy products <28 servings/week; “dairy products at pre-school: yes”—daily consumption of dairy products during pre-school period; “dairy products at pre-school: no”—lack of daily consumption of dairy products during pre-school period; “dairy products at school: yes”—daily consumption of dairy products during school period; “dairy products at school: no”—lack of daily consumption of dairy products during school period; “low BMD—bone mineral density < −1 SD”;“regular BMD—bone mineral density ≥−1 SD”.

A multi-dimensional analysis showed the relationship between the parameters and revealed two clusters (Figure 1). There was a high level of explained inertia in this model. Both dimensions explained together 58% of inertia: dimension 1%–33% of inertia and dimension 2%–25% of inertia. It means that the relation between BMD and risk factors of osteoporosis included in the model was moderately strong. In the S1 cluster, low BMD (below −1 SD) was associated with older aged women (≥50 years), lack of menstrual cycle. In the S2 cluster, regular BMD (≥−1 SD) was related to younger age of women (<50 years), presence of menstrual cycle, higher consumption of dairy products (≥28 servings/week) during adulthood and daily consumption of dairy products during childhood and adolescence. A strong relationship (cluster S2a) was detected for the consumption of dairy products at ≥28 servings/week and regular bone mineral density (BMD ≥ −1 SD). Both points representing consumption of dairy products at ≥28 servings/week and BMD ≥ −1 SD were separated by a short distance on the graph (in the same quadrant of the co-ordinate system). The points corresponding to non-consumption of dairy products during childhood and adolescence were weakly related to bone mineral density or other risk factors: they were situated on the graph at a larger distance from the S1 and S2 clusters, yet were close to each other.

## 4. Discussion

The studies showed that women’s bone mineral density to a large extent depended on the combined impact of dietary and non-dietary risk factors. The factors included, as well as age and the presence of the menstrual cycle, consumption of dairy products during adulthood and daily consumption of dairy products during childhood and adolescence. The single dietary risk factors related to bone mineral density were the daily consumption of dairy products during childhood and adolescence. The single dietary risk factors had a weaker impact on bone mineral density than non-modifiable confounders.

A strong relationship between the risk of osteoporosis and age and the menstrual cycle was confirmed [3,29]. The women with regular bone mineral density were younger and more of them had regular menstrual cycles. The probability of regular bone mineral density was four times higher in the women under 50 years of age and almost eight times higher in the women with regular menstrual cycles. The women who menstruated were under the influence of oestrogens, which support regular bone mineralization [6,30,31]. Non-menstruating women lack this protective effect of oestrogens and therefore menopause is an established risk factor for low bone density in women. The fastest loss of bone mass occurs in women in the first years after menopause due to rapid decrease in the level of oestrogens [3,16,30,31,32]. Collagen fibres degenerate with age and this process reduces the hardness of bones. Furthermore, bone loss overwhelms bone tissue formation [3,16]. Hence, age and menstrual cycle are strong independent risk factors for bone mineral density and osteoporosis.

The independent impact of single dietary risk factors on bone mineral density in the adult women was revealed for two dietary risk factors. The daily consumption of dairy products during pre-school and school period 2.4 to 2.7 times increased the probability of bone mineral density in the upper tercile in adult women. The relation between bone mineral density and consumption of dietary calcium above 400 mg/day in adulthood was weak–we stated a significance odds ratio only after adjusting for age but not after adjusting for age, menstruation and BMI together. The impact of calcium intake on bone density in later stages of life is less understood [10,13,16,33]. It is thought that in adulthood, the role of dietary calcium may be less and it may mainly influence the rate of bone loss. It is possible that our result followed a too low value of dietary calcium cut-off (400 mg/day). However, we could not use a higher cut-off because of the generally low dietary calcium intake level observed in women in our sample as well as in the Polish population [20,34,35]. The research should be continued with a greater number of women in the sample to explain the relation between dietary calcium and bone mineral density in adulthood and the point of good-fitted dietary calcium cut-off.

It is worth emphasizing that the impact of dietary factors on bone mineral density was connected to non-modifiable confounders. The relation was clear and is compatible with many papers [9,10,13,33]. The relationship between these risk factors was high since the explained variation of the system (inertia) was 58%. Our findings and the results by other authors indicate a multi-factorial aetiology of low bone mineral density and osteoporosis [10,14,15,16]. The number of risk factors for osteoporosis and bone fractures described in the literature exceeds 30 and this issue continues to be discussed and verified. It indicates a need for the simultaneous impact on many potential risk factors in the prevention of osteoporosis.

Interestingly, there is a relationship between the consumption of dairy products during adulthood and in the past and consequent benefits to bone health. The regular bone mineral density (S2 cluster) was revealed in younger and menstruating women who consumed more dairy products as adults and on every day in the past. The consumption of dairy products by the women during adulthood was relatively high since the established cut-off point was ≥28 servings/week. Such a consumption threshold was reached or exceeded by 89% of women with regular bone mineral density, *i.e*., six percentage points more than in the women with low bone mineral density. The consumption of dairy products in the past related to a daily intake of these products, regardless of their amount, because the collection of quantitative data from the period that extends 20–50 years in the past is virtually impossible in retrospective studies [24].

Our results indicate that the consumption of dairy products in childhood and adolescence had the largest and most positive impact on good bone health even if the level of intake in the past was unknown and the consumption of dairy products during adulthood was moderate. This conclusion is important for developing prevention programs and introducing preventive activities. It implies that childhood and adolescence are key periods for bone health. Firstly, this is the period of gaining peak bone mass [14,15]. Secondly, eating habits are established in this period. Numerous studies indicate that family and schoolmates during childhood and adolescence influence eating habits, consumption of food and human health in later stages of life [36,37,38,39]. Positive nutritional attitudes in parents and a good school environment facilitate and support eating behavior beneficial to health in children and adolescents. Prevention programs should thus aim at creating proper nutritional habits during childhood and adolescence and their reinforcement during adulthood.

Early formation of the habit of consuming dairy products daily may be critical to eating habits and bone health during adulthood [36,37]. Negative impact of low-calcium or non-dairy diets has been documented in Polish and foreign studies [40,41,42]. For instance, in Polish girls with diagnosed allergy to cow’s milk and treated with a non-dairy diet, bone fractures were found to occur four times more often than in girls who consumed an adequate amount of dairy products [41]. During childhood, nutrition depends on care-givers and is based on imitation. In later stages of life, food choices depend on a complex arrangement of many social, cultural, demographic, psychological and individual parameters such as age, sex, education, incomes, nutritional and health awareness, personal experiences, region and country-specific environment [43,44,45]. Generally, young people have a lower health risk perception than adults and seniors [46]. Therefore recalling diet-health awareness in order to improve the nutrition of young people can bring only small effects. This strengthens our earlier statement that in the young desirable eating habits should be formed by imitation and as a habitual behavior induced by the positive attitudes of parents and the good health impact of the school environment [36,37,38,39]. National health-nutrition policy should be focused on the early creation of good dietary habit in the first stage of life through the family and school environment.

The studies did not reveal any impact of non-dairy sources of calcium on bone mineral density. Our previous studies and some papers of other authors suggest a beneficial impact of taking calcium supplements and/or consumption of calcium-enriched food on bone health [29,47,48]. This paper did not focus on analyzing the impact on non-dairy calcium sources on the bones. For instance, during our studies we did not collect information on the amount of calcium consumed with supplements and/or calcium-enriched food. Hence, within the framework of this study, interpreting the impact of non-dairy calcium sources on risks for osteoporosis and bone fractures is difficult.

We found a relatively low level of dietary calcium in the adult women. The average level of dietary calcium was about 500 mg/day. Our results are consistent with the studies of many Polish authors [20,34,35]. In the WOBASZ studies it was found that adult Poles consumed on average 460 mg of calcium per day (less that 60% of DRI) [20]. In the national survey of households, the consumption of calcium by women aged 26–60 was 520 mg/day [31]. Higher consumption of calcium was recorded in Canadian women aged 50–70, *i.e*., on average about 750 mg/day [49]. Significantly higher calcium intake was reported in the prospective Swedish Mammography Cohort Clinical studies [10]. Swedish women in the lowest dietary calcium quintile consumed on average 698 mg/day, whereas the women in the highest quintile averaged 1389 mg/day. On this basis, the intake of dietary calcium by Polish women was assessed as relatively low.

The strengths and weaknesses of our research are as follows:

This relatively low level of dietary calcium is a weakness in our studies. Our results do not definitively conclude the occurrence of a relationship between dietary calcium and bone mineral density in relation to the recommended calcium intake by adult women. In Poland, the recommended intake (EAR) of calcium is set at 800 mg/day for women aged 31–50 and 1000 mg/day for women over 50 years of age [21]. It might indicate why our results cannot fully explain some of the opinions on the beneficial role of dietary calcium in reducing the risk of osteoporosis [3,9,10,13,50].

The dietary and densitometry studies were conducted on a sub-sample of 170 women that was not selected randomly from the general population. The dietary studies were carried out with 712 women and the consumption of dairy products was assessed. The analysis did not reveal any difference in the consumption of dairy products between the women from the total sample and the women from the sub-sample tested with densitometry. This increases the strength of drawing conclusions and provides a basis for formulating more general statements. The lack of randomization in the sample selection makes it difficult to refer the results to the general population, but it establishes a relationship between the consumption of dairy products and dietary calcium and the risk for low mineral density and osteoporosis. In this context, our findings provide some interesting information.

The use of a validated questionnaire on the frequency of food consumption for assessing the intake of dietary calcium by women during adulthood constitutes the strength of our studies [17]. The application of the consumption frequency method allows for an increase in the sample size in the dietary studies in comparison with food record or a 24-h interview [24]. Moreover, it creates an opportunity to evaluate food consumption over a longer time period. The questionnaire on the frequency of food consumption related to the consumption of dairy products during the six months preceding the study. It enabled a good assessment of the habitual consumption of dairy products and dietary calcium in the adult women.

## 5. Conclusions

It was found that bone mineral density in the adult women to a large extent depended on the combined impact of dietary and some non-modifiable confounders, which included the consumption of dairy products during adulthood, every day consumption of dairy products during childhood and adolescence as well as age and menstrual cycles. The influence of age and menstrual cycles on bone mineral density in the adult women was stronger than dietary factors and did not depend upon the presence of other risk factors. The impact of dietary factors on bone mineral density was mutually conditioned, and bone health depended on consumption of dairy products in childhood and adolescence and to a lesser extent in adulthood.

Several studies have found that the prevention of osteoporosis focusing only on a single risk factor may be insufficient for improving bone health. Our results show that there is a need for early creation of a dietary habit of daily consumption of dairy products in children and adolescents. Prevention programs should aim at establishing proper dietary behavior during childhood and adolescence and their reinforcement in the adult stages of life. It may be supposed that the daily consumption of dairy products during childhood and adolescence may reduce the negative effect of non-modifiable risk factors of osteoporosis in adulthood.

## Appendix A

**Table:** Comparison of dairy product consumption by women from the total sample and the sub-sample of women tested with bone densitometry (mean ± standard deviation; servings/week).

Dairy products	Total sample	Sub-sample	*p*-value for Kruskal-Wallis test
Sample size	712	170	
Milk	12.2 ± 7.0	12.4 ± 6.3	ns
Cheese	11.5 ± 5.9	11.2 ± 5.6	ns
Yoghurt	13.4 ± 6.5	13.4 ± 7.0	ns
Fresh cheese	5.4 ± 3.1	5.4 ± 3.0	ns
Cream	2.3 ± 1.3	2.2 ± 1.2	ns
Dairy products total	44.3 ± 14.8	44.5 ± 14.0	ns
Consumption of calcium-enriched food (% of the sample)	78	89	<0.05
Taking calcium supplement (% of the sample)	16	22	ns
Daily consumption of dairy products during pre-school period (% of the sample)	63	64	ns
Daily consumption of dairy products during school period (% of the sample)	57	61	ns

ns—non significant differences at *p* < 0.05.

## Appendix B

**Table:** The comparison of bone mineral density (BMD) between menstruation and no menstruation women or women with different age (mean ± standard deviation).

Variables	Total	Menstruation	No menstruation	29–39 years	40–49 years	50–59 years
Sample size	170	139	31	28	104	38
Age *** (years)	45.5 ± 5.8	44.1 ± 5.0	52.0 ± 4.6	37.0 ± 2.4	45.0 ± 2.8	54.4 ± 3.0
BMD (mg/cm^2^)	379 ± 59	382 ± 54	365 ± 76	372 ± 42	384 ± 56	370 ± 75
T-score BMD	0.58 ± 1.18	0.65 ± 1.08	0.30 ± 1.52	0.44 ± 0.83	0.68 ± 1.12	0.41 ± 1.50

Significance of differences between menstruation and no menstruation women: *** *p* < 0.001.
